# Efficacy and safety of colchicine for the treatment of osteoarthritis: a systematic review and meta-analysis of intervention trials

**DOI:** 10.1007/s10067-022-06402-w

**Published:** 2022-10-12

**Authors:** Ambrish Singh, Pablo Molina-Garcia, Salman Hussain, Alok Paul, Siddharth Kumar Das, Ying-Ying Leung, Catherine L. Hill, Debashish Danda, Jonathan Samuels, Benny Antony

**Affiliations:** 1 Menzies Institute for Medical Research, University of Tasmania, 17 Liverpool St, Hobart, Tasexermania 7000 Australia; 2 Virgen de Las Nieves University Hospital, Instituto de Investigación Biosanitaria ibs.GRANADA, Granada, Spain; 3 PROFITH (PROmoting FITness and Health Through Physical Activity) Research Group, Department of Physical Education and Sports, Faculty of Sport Sciences, University of Granada, Granada, Spain; 4 Czech National Centre for Evidence-Based Healthcare and Knowledge Translation (Cochrane Czech Republic, Czech EBHC: JBI Centre of Excellence, Masaryk University GRADE Centre), Institute of Biostatistics and Analyses, Faculty of Medicine, Masaryk University, Brno, Czech Republic; 5 School of Pharmacy and Pharmacology, University of Tasmania, Hobart, Tasmania Australia; 6 Era’s University, Lucknow, India; 7 Department of Rheumatology and Immunology, Singapore General Hospital, Singapore, Singapore; 8 Rheumatology Unit, The Queen Elizabeth Hospital, Woodville South, South Australia Australia; 9 Discipline of Medicine, University of Adelaide, Adelaide, South Australia Australia; 10 Department of Clinical Immunology and Rheumatology, Christian Medical College, Vellore, India; 11 Department of Medicine, Division of Rheumatology, NYU Grossman School of Medicine, New York, New York USA

**Keywords:** Calcium pyrophosphate, Chondrocalcinosis, Colchicine, Osteoarthritis, Osteoarthritis knee

## Abstract

**Objective:**

Colchicine, an approved treatment for gout, has been trialed in many diseases including osteoarthritis (OA) due to its anti-inflammatory effects. However, its efficacy and safety remain unclear in OA. This systematic review and meta-analysis evaluated the efficacy and safety of colchicine for the treatment of OA.

**Methods:**

PubMed, Web of Science, Scopus, and Cochrane Central were searched from inception through September 2022. Two reviewers independently screened for randomized controlled trials (RCTs) comparing colchicine with placebo or other active comparators for the treatment of OA (knee, hand, or hip OA), extracted data, and performed Cochrane risk of bias assessments.

**Result:**

Nine RCTs for the knee OA and one for the hand OA were identified, consisting of 847 patients (429 in colchicine arms, 409 in control arms). The studies were conducted between 2002 and 2021 with follow-up periods ranging from 2 to 12 months, in India, Iran, Turkey, Australia, Singapore, and Iraq. Moderate-quality evidence showed no clinically important pain reduction with colchicine compared to control (standardized mean difference [SMD], 0.17; 95% confidence interval [CI], − 0.55, 0.22). Moderate-quality evidence showed no improvement in function with colchicine compared to control in knee OA patients (SMD, − 0.37; 95% CI, − 0.87, 0.13). Colchicine showed an acceptable safety profile with AEs/SAEs comparable to control.

**Conclusion:**

Current evidence does not suggest a benefit of colchicine in reducing pain and improving physical function in the overall cohort of hand/knee OA patients. Future trials should focus on the subgroups of OA patients with local or systemic inflammation and/or mineralization who might benefit from colchicine.

**Graphical abstract:**

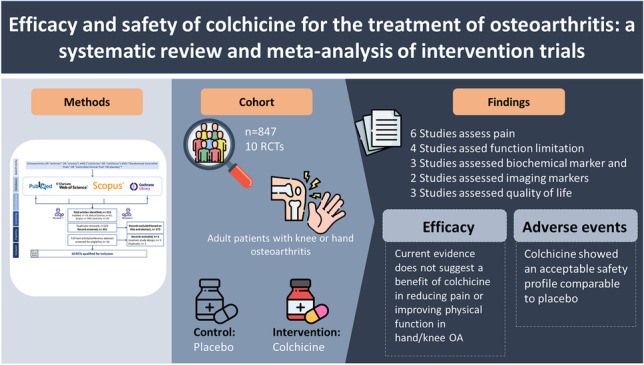

**Supplementary information:**

The online version contains supplementary material available at 10.1007/s10067-022-06402-w.

**Table:** 

**Key Points** •*Colchicine is an approved treatment for gout that has been trialed in many diseases including osteoarthritis (OA) due to its anti-inflammatory effects. However, the benefit and harms of colchicine in OA remain unclear.* • *Current evidence from randomized control trials does not suggest a benefit of colchicine in reducing pain and improving physical function for the treatment of OA patients.* • *Future trials of colchicine in OA should focus on the subgroups of OA patients with local or systemic inflammation and/or mineralization who might benefit from colchicine.*	

## Introduction

Osteoarthritis (OA) is a common and chronic degenerative joint disorder that mainly affects the knee joints with the hip, hand, and spine being other common sites. The global burden of OA increased further due to the aging population and the rising prevalence of obesity [1]. As per the Global Burden of Disease (GBD) Study 2019, OA is ranked 16th among the causes of global years lived with disability (YLD) [2] [2–4]. Despite the high prevalence and global impact of OA, there are no disease-modifying drugs approved for the treatment of OA that can arrest, slow, or reverse the progression of structural damage of the joint. [1, 3].

Inflammation is an established pathological feature of OA and numerous proinflammatory cytokines (such as interleukin (IL)-6, monocyte chemoattractant protein 1, vascular endothelial growth factor, interferon γ–induced protein, and monokine) have been identified in the OA joint. [4] The progressive destruction and remodeling of the joint in OA have been linked with proinflammatory factor-mediated stimulation of matrix-degrading enzymes, such as matrix metalloproteinases (MMPs) [4]. Furthermore, the radiographic severity of knee OA has been found to be associated with proinflammatory cytokines such as IL-18 and IL-1β [5, 6]. Interestingly, the IL‐18 and IL-1β are also produced by macrophages through uric acid crystal-induced inflammasome assembly during gout attacks [7]. Investigators have found a positive association between uric acid levels in synovial fluid and the severity of knee OA [5, 6], similarly monosodium uric acid (MSU) can accumulate in joints and influence the synthesis of inflammatory cytokines. [8] Notably, inflammation in knee OA is often accompanied by the observable presence of calcium pyrophosphate dihydrate (CPPD) crystals leading to the production of IL‐1, an essential mediator of cartilage breakdown in OA [9]. These factors implicate the innate immune system and subsequent uric acid production in the pathology and progression of knee OA [4].

Colchicine is a naturally occurring alkaloid derived from several plants from the Colchicaceae family, such as *Colchicum autumnale* [10]. Colchicine had demonstrated anti-inflammatory and antifibrotic activity and has been used over decades for gout, familial Mediterranean fever, pericarditis, and other inflammatory and dermatologic disorders [10, 11]. Our group previously reported that colchicine provided significant symptomatic relief in patients with knee OA [9, 12], while other investigators have found similar results. [13] Two systematic reviews have explored the effect of colchicine on OA but focused only on knee OA, did not use the standardized mean difference (SMD) approach to meta-analyze the results, and did not evaluate the biomarkers (imaging and biochemical markers) and quality of life (QoL) outcomes. These studies report conflicting findings and conclude that colchicine does not improve symptoms, although it reduced inflammation in knee OA patients [14–16]. While the exact mechanism of action of colchicine for the treatment of OA is still to be explained, several studies have explored colchicine as a possible option for the treatment of OA [9, 12–15, 17, 18] and there are few ongoing studies of colchicine as a treatment for OA [19, 21]. Hence, the aim of this systematic review and meta-analysis was to assess the efficacy and safety of colchicine for the treatment of patients with OA.

## Methods

### Search strategy, selection, and data extraction

This review was guided by the Preferred Reporting Items for Systematic Reviews and Meta‐Analysis (PRISMA) [22], and we followed our protocol registered and published at medRxiv (DOI number: 10.1101/2020.11.20.20226589) and reported following the Preferred Reporting Items for Systematic Reviews and Meta-Analyses (PRISMA) guidelines. [23] Four bibliographic databases—PubMed, Web of Science, Scopus, and the Cochrane Central Register of Controlled Trials—were searched from inception till September 2022, with English language restriction. The search strategy included a combination of Medical Subject Heading (MeSH) such as (Osteoarthritis OR “arthrosis” OR “artrosis”) AND ("colchicine" OR "colchicina") AND (“Randomized Controlled Trials” OR “Controlled Clinical Trial” OR placebo) (Appendix 1).

Hand-searching of abstracts from the last 2-year conference proceedings of key international associations involved in OA and rheumatology research, such as the American College of Rheumatology (ACR), European League Against Rheumatism (EULAR), and Osteoarthritis Research Society International (OARSI), was performed to identify recent addition RCTs that may not be captured in databases search. Information on the latest clinical trials on colchicine was obtained from principal investigators of the major clinical trials of colchicine for OA, who are the authors in this systematic review. The articles were screened first based on their title and abstracts and then based on full-text for their inclusion as per prespecified eligibility criteria by three researchers independently (AS, PMG, and SH).

PICOS strategy was used to determine the eligibility of studies based on population, interventions, comparators, outcomes, and study design. The population was adults older than 18 years, of any sex, and diagnosed with OA according to the American College of Rheumatology criteria or similar approaches. [24] Intervention was an oral alone dose of colchicine or in combination with other conventional drugs such as Paracetamol or NSAIDs. The control was placebo or active pharmacological intervention (including NSAIDs) but not non-pharmacological intervention (such as yoga, physiotherapy, exercises, occupational therapy, medical devices, acupuncture, behavioral interventions, education, or surgery). The efficacy outcomes of interest consisted (1) change in OA-associated pain and physical dysfunction evaluated using Western Ontario and McMaster Universities Osteoarthritis Index Score (WOMAC), visual analog scale (VAS), or other patient-reported outcome (PRO) instruments; (2) imaging biomarkers such as radiographic (X-ray) and/or MRI structural measures; (3) biochemical markers such as serum levels of MMP3, HA, etc. (4) medication change; and (5) QoL assessed using EQ-5D, SF-6D, or other multi attributable utility instruments. Safety outcomes included adverse effects (AEs) and serious adverse effects (SAE) reported with colchicine. When more than one pain measure was reported, we considered the pain outcomes in the following order: VAS, pain subscale of WOMAC, NRS, and any other patient-reported pain measures. Lastly, the study design was randomized control trials, quasi-randomized, and non-randomized control trials with blinded or non-blinded designs. Studies (or abstracts) written in languages other than English or Spanish were excluded.

The related data, such as the study design, population characteristics, intervention/comparator details, and change in efficacy and safety outcomes, were extracted using a pre-designed data extraction MS Excel® sheet. Two investigators (AS and PMG) performed data extraction, and any discrepancy at the screening and data extraction stages was resolved by mutual discussion or arbitration by the senior investigator (BA).

### Quality assessment

We evaluated the bias risk of the included studies according to version 1 of the Cochrane risk-of-bias tool for randomized trials (RoB 1). [25] The RoB 1 is structured into a fixed set of domains of bias, focusing on different aspects of trial design, conduct, and reporting. Seven domains were evaluated following the *Cochrane Handbook* V.5.1.0, Chapter 8.5: (1) random sequence generation, (2) allocation concealment, (3) blinding of participants and personnel, (4) blinding of outcome assessment, (5) incomplete outcome data, (6) selective reporting, and (7) other biases. Two investigators (AS and SH) evaluated the quality; any difference of opinion was resolved by discussion or arbitration by the senior investigator (BA). The final assessment is classified as “Low,” or “High” risk of bias or can express “Some concerns”. (3).

### Data synthesis and analysis

The mean change in continuous outcomes, such as pain and symptoms, were used to estimate the pooled effect estimates. The data on change from baseline to follow-up was calculated as the arithmetic difference between baseline and longest reported follow-up. The reported standard deviations (SD) were used, if not reported, were calculated using reported standard error (SE) or confidence intervals (CI). The change-from-baseline SD was calculated using the methods described in the *Cochrane Handbook for Systematic Reviews of Interventions* (Chapter 6; Sect. 6.5.2.8) [26], and a conservative correlation coefficient value of *r* = 0.5 was used [27, 28]. Since the included studies had assessed the outcome measures using different scales (e.g., WOMAC and KOOS to evaluate osteoarthritis-associated dysfunction), we standardized the results to a uniform scale using standardized mean difference (SMD) to enable the pooling of data in the meta-analysis. (3) One study [12] reported the results only in plots, and we extracted the data through the WebplotDigitizer software [29], which has demonstrated an excellent validity and reliability in extracting graphed data [30]. The statistical heterogeneity was assessed as per *Q* statistics (*p* < 0.05 was considered heterogeneous), and *I*^2^ statistic (*I*^2^ > 50% was deemed to be heterogeneous) [31]. A meta-analysis of the included studies was performed using the generic inverse variance random-effects model using Review Manager 5 (RevMan 5.4) [32]. The sensitivity analysis was performed using the leave-one-out method. The funnel plot was not developed since the meta-analysis included less than ten studies.

## Results

**Fig. 1:**
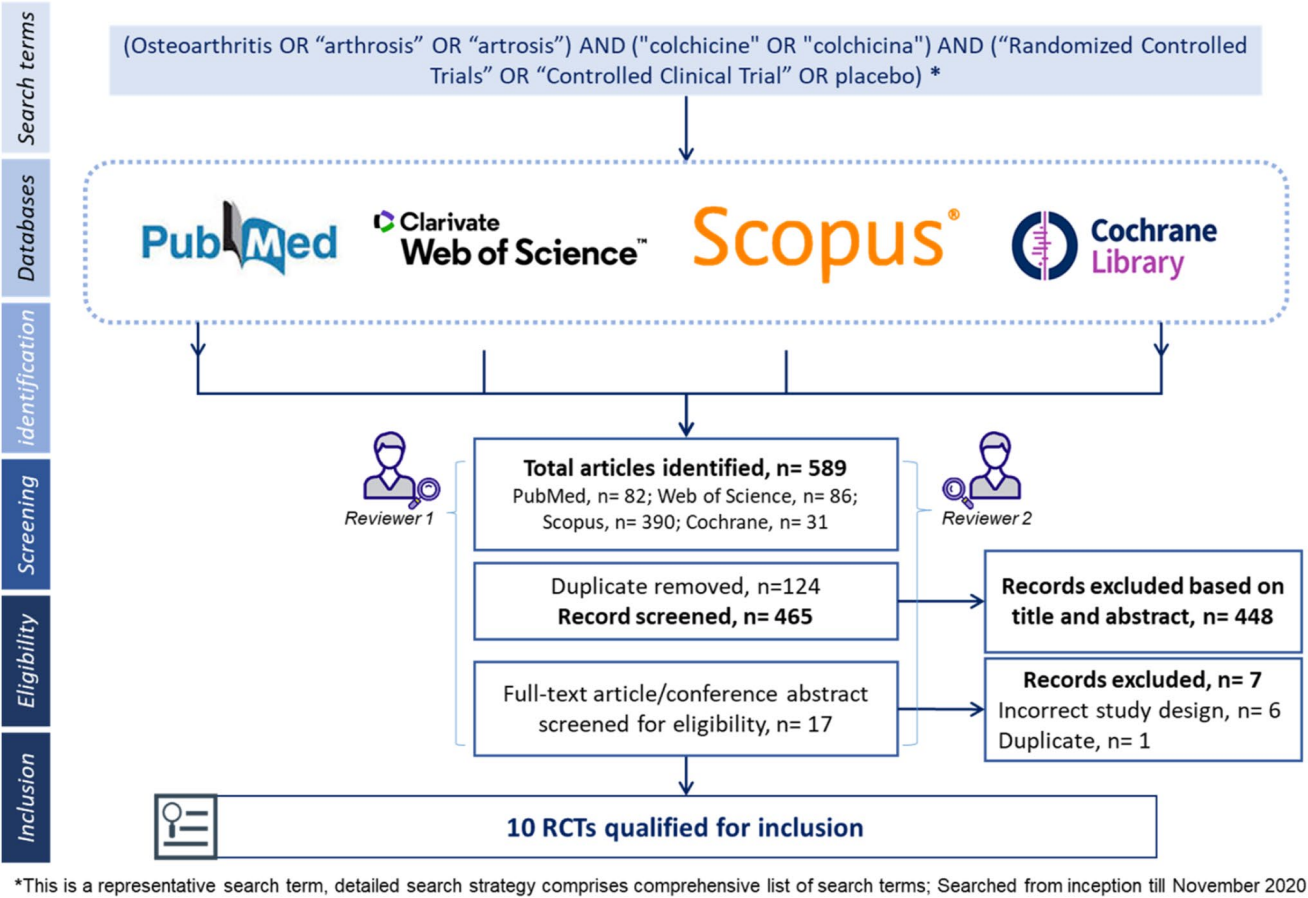
Flow chart describing method and inclusion of studies

The literature search and screening process are shown in the PRISMA flow diagram (Supplement Fig. 1). The exhaustive literature search retrieved a total of 589 articles from four databases. Overall, 82 articles were sourced from PubMed, 86 from Web of Science, 390 from Scopus, and 31 from Cochrane library. No additional articles were identified by hand-searching. After duplicate removal, 465 articles were screened, and ten articles were included in this systematic review with 455 articles being excluded due to reasons such as incorrect study design, and duplicates (Fig. 1).

### Characteristics of the included studies

A total of 847 patients were enrolled in ten included RCTs, with a total of 429 in the interventional (colchicine) arms and 409 in the control arms. Nine of the ten studies had enrolled patients with knee OA, and one with hand OA, while none of the studies assessed colchicine in hip OA. Four of the studies compared colchicine with placebo [13–15, 17], and the remaining studies had an (NSAID) in both interventional and control arms. The studies were conducted between 2002 and 2020, with three in India, two in Iran and Turkey, and one each in Australia, Singapore, and Iraq. The follow-up period ranged between 2 and 12 months. For two of the included studies, data was only available from an abstract of a scientific meeting; one was published in non-English (with only an abstract in English) and the other was published as a conference abstract. The full data from these studies were not available despite emailing the authors [33, 34]. Table 1 provides the characteristics of the included studies.

**Table 1:** Key description of the included studies

Author (year), country, trial name, trial registration number	Study design	Participants					Outcome			Intervention			Control group	Conclusion
		Sample (IG vs CG)	Age range	Women, *n* (%)	OA type; diagnosis	Presence of gout/crystal arthropathy	Key outcomes	Assessment method	Adverse effects	Intervention group	Other drugs	Duration		
Das SK (2002) AC, India, ND, ND	RCT, double-blind, single-center	36 (19 vs. 17)	40–75	77.8	Knee OA, ACR, WOMAC, RF (-ve)	CPPD crystal in 8 patients. IG: 5; CG: 3	Knee and overall pain, global assessment, clinical health	VAS, WOMAC, ModHAQ	Yes	COL 0.5 mg bd + nimesulide 100 mg	Tramadol 50 mg	5 months	Placebo + nimesulide 100 mg	COL provided greater symptomatic benefit
Das SK (2002) OC, India, ND, ND	RCT, double-blind, single-center	39 (19 vs. 20)	40–75	68.4	Knee OA, ACR, inflammation symptoms, RF(-ve)	Cartilage calcification (chondrocalcinosis) seen in 38%. CPPD crystals in 74% of patients IG: 15; CG: 14	Knee and overall pain; global assessment; Clinical health; Drugs intake	VAS and Doyle’s index, KGMC (modified WOMAC) and physicians’ assessment, number of drugs	Yes	COL 0.5 mg bd + piroxicam 20 mg od	+Piroxicam 20 mg od	5 months	Placebo + piroxicam 20 mg od	COL produced significantly greater symptomatic benefit
Aran S (2011), Iran, ND, ND	RCT, double-blind, single-center	58 (29 vs. 29)	49–78	100	Knee OA, ACR, OKS	No patients had CPPD	Global assessment (self-reported and physician); Drugs intake	VAS knee pain, number and dose of drugs	Yes	COL 0.5 mg bd	Acetaminophen < 2 g/day	3 months	Placebo (Vitamin B6)	Efficacy and safety of COL was affirmed
Ediz L (2012)ǂ, Turkey, ND, ND	RCT, open-label, single-center	74 (33 vs. 32)	40–75	70.3	Knee OA, ND	No patients had CPPD or gout	Pain and function	VAS and WOMAC	Yes	COL 1.5 g od + acetaminophen 3 g od	Acetaminophen 3 g od	6 months	Acetaminophen 3 g od	Addition of COL to acetaminophen produced significantly greater symptomatic benefit in knee OA
Erden M (2012), Turkey, ND, ND	RCT, open-label, single-center	110 (60 vs. 50)	50–63	60	Knee OA, ACR, microscopy	Participants with CPPD crystals were included. Patients with gout were excluded	Pain and function, oxidant/antioxidant status	VAS and WOMAC, blood sample	No	COL 1.5 g od + paracetamol 3 g od	ND	6 months	Acetaminophen 3 g od	Addition of COL to paracetamol produced significantly greater symptomatic benefit than paracetamol alone in knee OA
Amirpour A (2016), Iran, ND, IRCT2015071623240N1	RCT, double-blind, single-center	62 (32 vs. 30)	35–75	98	Knee OA, ACR or radiographic	No patients had CPPD or gout	Pain, functional disability, clinical health, biomarker	VAS scale, WOMAC, HAQ	ND	COL 0.5 mg bd	ND	4 months	Placebo	COL reduced pain and improved physical function
Srivastava R (2018)*, India, ND, ND	Non-RCT, non-blinded, single-center	150 (75 vs. 75)	ND	ND	Knee OA, ND	No confirmation on CPPD presence. No presence of MSU crystal. No patients had gout	Biomarker (COMP)	VAS, serum COMP by ELISA	ND	COL 0.5 mg bd + paracetamol 1 g td	Acetaminophen 1 g td	1 year	Acetaminophen 1 g td	The COMP level was stable in colchicine group, indicating that it may act as a disease-modifying agent
Leung YY (2018), Singapore, COLKOA, NCT02176460	RCT, double-blind, single-center	109 (54 vs. 55)	21–79	70.4	Knee OA, ACR and radiography	No patient has gout/MSU crystals No confirmation on CPPD presence	Pain, stiffness and function, biomarkers	WOMAC, VAS and OARSI-OMERACT; blood, synovial fluid, and urine samples	Yes	COL 0.5 mg bd	Acetaminophen < 2 g/day	4 months	Placebo	COL did not improve the symptom in knee OA, however reduced inflammation and high bone turnover biomarker
Salman S (2020)†, Iraq, ND, ND	RCT, double-blind, single-center	150 (80 vs. 70)	ND	ND	Knee OA, ND	No confirmation on CPPD presence or gout	Pain, stiffness, and function	WOMAC	No	COL 0.5 mg + paracetamol 500 mg bd	ND	ND	Acetaminophen 500 mg bd	Addition of COL to paracetamol produced longer symptomatic improvement in knee OA compared to paracetamol alone
Davis CR (2020), Australia, COLAH, ACTRN12617001524381	RCT, double-blind, single-center	59 (28 vs. 31)	40–80	84.4	Hand OA, radiography (X-ray)	No confirmation on CPPD presence. No patients had gout	Hand pain, swollen joint, grip strength, hand function and pain, inflammatory proteins	VAS, Medical exam, hand dynamometer, MHQ, blood sample	Yes	COL 0.5 mg bd	ND	3 months	Placebo	COL was not effective in reducing pain tender and swollen joint count or increasing grip strength in symptomatic hand OA

ACR: American College of Rheumatology; AC: Arthritis Care & Research; IG: intervention group (colchicine); CG: control group; COL; colchicine; COMP: cartilage oligomeric matrix protein; ELISA: enzyme-linked immunosorbent assay; HAQ: Health Assessment Questionnaire; MHQ: Michigan Hand Questionnaire; KOOS: Knee Injury and Osteoarthritis Outcome Score; ModHAQ: Modified Clinical Health Assessment Questionnaire; MSU: monosodium uric; ND: Not discussed; NSAIDs: Nonsteroidal anti-inflammatory drugs; OC: Osteoarthritis and Cartilage; OKS: Oxford Knee Score; RCT: randomised control trial; RF: rheumatoid factor

^†^ published as abstract;

ǂ data captured based on English language abstract

^*^No pain outcomes were included as it is a non-randomised trial reporting biochemical markers relevant to OA

### Assessment of quality and risk of bias

**Fig. 2:**
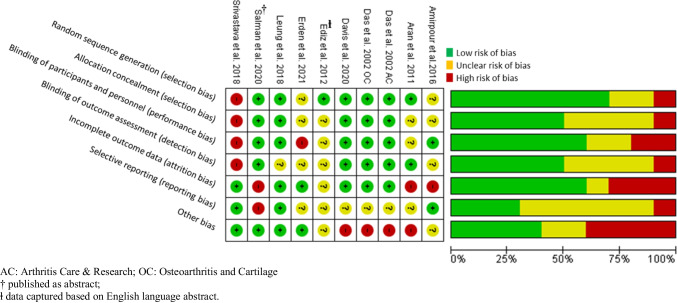
Cochrane risk of bias assessment

The overall risk of bias in included studies was high, with five trials assessed being low quality, according to the Cochrane RoB I tool (Fig. 2). Seven of the included RCTs were assessed as having a high or unclear risk of bias for selective reporting, and three were assessed as having a high risk for incomplete outcome data reporting either due to loss of follow-up or lack of intention-to-treat (ITT) data reporting. Four trials were assessed as low quality due to a smaller sample size under “Other bias” category.

### Effect on OA pain

**Fig. 3:**
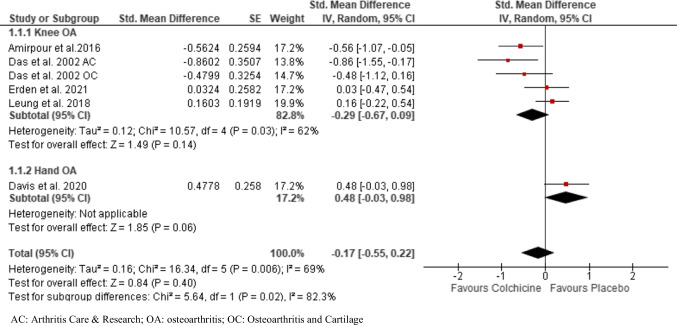
Pooled standardized mean difference for change in osteoarthritis-associated pain

Overall, six trials constituting 212 participants in the colchicine groups and 203 participants in the control groups were included in the analysis of pain in knee/hand OA patients [9, 12, 14, 15, 17, 18] (Fig. 3). Five RCTs assessed pain in knee OA [9, 12, 14, 17, 18] and one in hand OA [15]. All six trials used PRO instruments, mainly pain VAS and WOMAC. We found moderate-quality evidence that colchicine had no clinically important pain reduction compared to control in knee/hand OA patients (SMD, − 0.17; 95% CI, –0.55 to 0.22). The effect size was modest, but it was still statistically non-significant (SMD 0.29, *p* = 0.14) when knee OA studies were pooled together. An *I*^2^ statistic of 69% indicated a substantial degree of statistical heterogeneity. Similar results were observed in sub-group analysis including studies comparing colchicine with placebo (Supplemental Fig. 2).

The leave-one-out sensitivity analysis indicated that the pooled estimates were not dependent on any single study (Supplemental Table 3).

### Effect on physical function

**Fig. 4:**
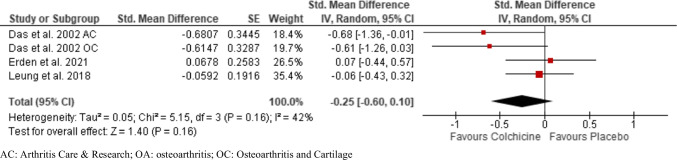
Pooled standardized mean difference for change in knee osteoarthritis-associated dysfunction

Four studies reported WOMAC function limitation in knee OA, consisting of 152 participants in the colchicine groups and 142 participants in the control groups [9, 12, 14, 18]. (Fig. 4). Moderate-quality evidence with pooled SMD: − 0.25 (95% CI, − 0.60 to 0.10) showed that colchicine had no improvement in dysfunction compared to control in patients with knee OA. An *I*^2^ statistic of 42% indicated a moderate degree of statistical heterogeneity among included studies.

Leave-one-out analysis for the pooled analysis showed that the pooled estimate was sensitive to the omission of Erden et al. (Supplemental Table 4).

### Biomarkers

#### Osteoarthritis-related biochemical markers

Three studies assessed biochemical markers of OA, including the one-hand OA study [14, 15, 35]. In knee OA patients, Srivastava et al. reported a significant increase in serum COMP levels from 2 months to 1 year of follow-up in the paracetamol-alone group, while it remained stable in the colchicine group [35]. In another study in knee OA patients, serum levels of hs-CRP and synovial fluid levels of CTX-I were significantly reduced in the colchicine but not the placebo-treated arm over a 4-month follow-up [14]. Davis et al. study in hand OA patients reported no significant difference between groups for serum CRP, CK, or liver enzymes (ALT and AST). [15].

#### Osteoarthritis-related imaging markers

Only two studies evaluated OA-related imaging marker outcomes [14, 15]. Davis et al. reported no significant difference between the group for ultrasound-assessed synovitis grade in hand OA patients. [15] Similarly, Leung et al. reported no significant difference in MRI-assessed effusion size or infrapatellar synovitis between treatment arms in knee OA patients in a small random subset of participants [14].

The main conclusions on biomarker outcomes (i.e., biochemical and imaging markers) are summarized in Supplement Table 1.

#### Osteoarthritis-related quality of life

Three studies in knee OA patients, 92 each in the colchicine and control arms, assessed QoL using the generic PRO instruments: the Health Assessment Questionnaire (HAQ), 36-Item Short Form Survey (SF-36), and modified HAQ (ModHAQ) [9, 12, 14]. The ModHAQ is a non-validated modification of a scale used at All India Institute of Medical Sciences, New Delhi, India [9, 12]. Two studies by Das et al. reported a significant improvement in ModHAQ score in the colchicine group compared to the control [9, 12]. However, Leung et al. found no statistically significant improvement in HAQ or SF-36 (PCS and MCS) scores [14]. A summary of the main conclusions on QoL can be found in Supplemental Table 2.

### Adverse events

**Fig. 5:**
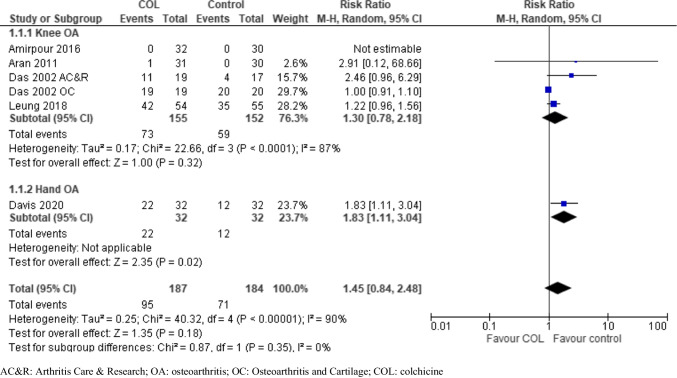
Adverse effects of colchicine

Among the included ten studies, six reported safety outcomes [9, 12–15, 17]. Most AEs reported were transient and mild-to-moderate severity. Notable AEs such as diarrhea, myalgia, and elevated creatinine phosphokinase (CPK), which are known to be related to colchicine, occurred at a higher rate in the colchicine group. No SAEs were reported by any of the included studies. The pooled analysis revealed no significant difference in AEs (risk ratio [RR]: 1.45; 95%CI 0.84, 2.48) between colchicine and placebo/active comparators (Fig. 5).

## Discussion

To the best of our knowledge, this is the most comprehensive systematic review and meta-analysis assessing the efficacy and safety of colchicine for the treatment of OA. We did not find evidence to suggest a beneficial effect of colchicine in reducing pain and improving physical function in hand/knee OA patients compared to the control groups. Furthermore, pooled evidence—that was of moderate quality—suggested that colchicine demonstrated only a small (SMD: 0.3) and statistically non-significant improvement in pain and physical dysfunction compared to the control group for patients with knee OA. [36] Limited evidence was reported assessing the effect of colchicine on biochemical markers [14, 15, 35] and imaging markers [14, 15] in knee OA patients. Two of the three available trials studying QoL demonstrated superior improvements with colchicine treatment compared to control; however, both studies used non-validated generic PRO instruments not specifically developed for knee OA [9, 12]. The overall safety profile of colchicine was acceptable with AEs/SAEs comparable to control [9, 12–15, 17].

Results from our meta-analysis reveal no superior effects of colchicine treatment on pain and physical function in OA patients. There are several factors that could be explaining these results. First, the majority of the studies reporting a favorable effect of colchicine in OA were conducted with a small sample size (approx. 80 participants) [9, 12, 13, 17, 34, 37]. A systematic review on the efficacy of colchicine for the treatment of knee OA reported a significant reduction in knee pain based on four small sample-sized trials (all conducted in Asia and the middle east) [38]. However, in a recent RCT by Leung et al., colchicine did not improve knee symptoms—despite showing a favorable reduction in systemic inflammatory markers and high bone turnover biomarkers [14]. Similarly, a recent RCT by Davis et al. reported that colchicine did not improve pain, reduce tender or swollen joint counts, or increase grip strength in patients with symptomatic hand OA [15]. However, this study’s cohort was not enriched for hand OA patients having active inflammation, who may have a better chance of responding to colchicine [37]. A second factor contributing to a lack of efficacy is the lack of studies that have an enriched subpopulation of knee OA patients having evidence of local/systemic inflammation, who may have a better response to colchicine. However, Das et al. studies had included patients with at least two of the four clinical signs of inflammation (warmth over the joint area, joint margin tenderness, synovial effusion, and soft tissue swelling around the knee) and reported positive effects of colchicine on knee symptoms [9, 12]. Recent trials in OA are moving towards precision medicine; thus, future trials in colchicine could focus on studying the effect of colchicine on those phenotype/subpopulations of OA that might be more responsive to colchicine. The recent study, which does not qualify for inclusion, compared colchicine with physical therapy and found physical therapy to be more effective than colchicine in reducing symptoms of knee OA. [39]. Currently, there are two ongoing clinical trials of colchicine one in knee OA and one in hand OA each [19, 21]. The interim blinded results of the ongoing CLOCAK trial were recently reported as a conference abstract; however, it did not qualified inclusion in our study due to the unavailability of results reported for the intervention and treatment arm separately [21] (Supplemental Table 5).

A third concern is that the average intervention duration was less than 5 months. This time period may be adequate to demonstrate symptom-relieving response, and this might not be enough time to show the structural change that may lead to positive effects in OA disease. For instance, Srivastava et al. reported no significant changes in the marker of cartilage degradation at 2 months, but they found that the control group increased several biomechanical markers (i.e., serum COMP) at 1 year, while no such disease progression was reported in the colchicine group. Future studies should include a longer follow-up period, at least 1 year, to determine the long-term effects of colchicine in OA.

Colchicine is thought to act in OA via its tubulin disruption mechanisms and downregulation of multiple inflammatory pathways [6, 40]. Colchicine prevents the crystallization of articular cartilage, a common symptom in end-stage OA [41, 42]. The crystallization of calcium pyrophosphate deposition (CPPD) is frequently seen in severe OA and is often associated with acute and chronic inflammation in the joint. For instance, CPPD deposition is frequently seen in advanced knee OA through X‐ray imaging and is often associated with acute and chronic inflammation in the joint. The descriptive term indicating the presence of gross CPPD within knee cartilage (i.e., both hyaline and fibrocartilage) is chondrocalcinosis, and 25–30% of knee specimens harvested at the time of surgery have chondrocalcinosis [43]. The majority of the studies included in our systematic review excluded patients with any evidence of CPPD, and two studies exclusively included patients without CPPD [13, 17] Amirpour et al. [17] and Aran et al. [13] reported positive effects of colchicine on pain and functionality in knee OA patients (excluded patients with evidence of CPPD), while Erden et al. did not find superior improvements of colchicine on either pain or functionality in OA patients with CPPD [18]. These results might suggest that colchicine treatment is more effective in preventing inflammation (e.g., crystal-induced inflammatory cytokines) than reducing crystallization in OA patients. However, the two studies assessing OA-related imaging markers found no significant reduction in local joint inflammation signs (i.e., synovitis grade, effusion size, or infrapatellar synovitis) in the colchicine group compared to placebo despite showing a reduction in the systemic inflammatory markers [14, 15]. However, the sample size of these studies are smaller, and thus, larger sample size RCTs are required to confirm the effectiveness of colchicine in patients with OA.

Previous evidence shows that colchicine prevents crystal‐induced inflammation in other rheumatic diseases such as gout and pseudogout [40]. These diseases are associated with MSU or CPPD crystal deposition in joints and periarticular tissues [44] that engage the caspase‐1‐activating NALP3 (also called cryopyrin) inflammasome, resulting in the production of active IL‐1β and IL‐18 [7]. These pathways are crucial in OA progression and are key features of OA pathogenesis [7]. The inflammation is attenuated by colchicine through the phosphorylation of tyrosine created by microcrystals. Furthermore, colchicine is known to reduce the formation of IL-1 levels in crystal arthritis, and IL-1 has been shown to be correlated with serum COMP levels in OA [35, 45, 46]. Within our review, we found only three studies that assessed OA-related biochemical markers. Srivastava et al., in the 1-year follow‐up study, found an increase in serum cartilage oligomeric matrix protein (COMP) levels from 2 months to 1 year in the placebo group, whereas COMP levels remained unchanged in the colchicine group. This finding could be signifying the lack of uncoupling of collagen from aggrecan in the colchicine group and hence reducing disease progression [35]. Leung et al. reported a significant reduction in mean levels of serum hs-CRP proinflammatory and SF CTX-I cartilage degradation biomarkers in the colchicine compared to the placebo [14]. Overall, these results suggest that colchicine reduces the concentration of key proinflammatory and cartilage degradation markers in OA, although additional trials are needed to corroborate that statement with quantitative meta-analyses.

Colchicine has a long history of use to treat acute flares in gout and is generally regarded as having a good safety profile, especially in low doses [47, 48]. A recent systematic review showed that the common adverse events with colchicine use are limited to diarrhea and gastrointestinal events and SAEs, including the liver and hematological changes, muscle toxicity, and neuropathy, are rare in clinical trials [49]. Our meta-analysis reveals a non-significant trend of higher AEs associated with colchicine, a fact that should be considered in its prescription. The majority of the included studies in this systematic review used a 0.5 mg twice-a-day dose of colchicine, which falls within the range of recommended dose for treatment of CPPD and gout [50]. In line with the previous evidence, the studies reported AEs to be transient and of mild-to-moderate severity and higher dosages of colchicine, although might provide more effective results, would likely enhance AEs. However, it is known that using the traditional regimens of 1 mg loading dose and 0.5 mg maintenance every 2 h increases the AEs/SAEs considerably [51]. Furthermore, the AEs that are known to be related to colchicine may have also impacted the concealment of allocation.

The main strengths of this systematic review consist of a published protocol-oriented approach, extensive database search, and the application of suitable statistical techniques to pool the effect estimates. However, there are some limitations that should be mentioned. Firstly, all included studies used self-perceived questionnaires to evaluate both pain and physical functioning, which could be introducing bias due to inaccurate reporting. A recent systematic review synthesis of the main objective method to evaluate pain experience (e.g., electro neurophysiological tests and mechanical or thermal sensors to establish pain threshold) [52]. Furthermore, accelerometers and objective assessment of physical function have been recommended to provide a more valid indicator of the physical activity profile and functional level. [53, 54]. We claim future trials including objective measures of pain and functionality to obtain more valid results on the effectiveness of colchicine interventions. Secondly, the methodological quality (smaller sample size, blinding, incomplete/selective reporting of results) of most of these previous studies was not sufficient to draw definitive conclusions. Furthermore, infrequent data reported in primary papers limited the scope for detailed subgroup analysis, and publication bias assessment, using a funnel plot, was not possible due to less than ten studies included in the meta-analysis. Thirdly, in the majority of studies, we found heterogeneous population of patients with OA since did not consider different phenotypes such as imaging or inflammatory markers. Lastly, due to the insufficient data in some trials, SD values were imputed; however, we used the prescribed methods and assumptions [3, 26–28].

## Conclusion

Current evidence does not suggest a benefit of colchicine in reducing pain and improving physical function in hand/knee OA patients. Future trials that are sufficiently powered should focus on the subgroups of OA patients with local or systemic evidence of inflammation and/or mineralization that may benefit from colchicine.

## Supplementary information

Below is the link to the electronic supplementary material.

Supplementary file1 (DOCX 227 KB)
