# Supplementary material for: Efficacy and safety of colchicine for the treatment of osteoarthritis: a systematic review and meta-analysis of intervention trials
Source: Clin Rheumatol. 2022 Oct 12;42(3):889–902. doi: 10.1007/s10067-022-06402-w (PMC9935673; doi:10.1007/s10067-022-06402-w)
Supplement: Supplementary file 1 — Supplementary file1 (DOCX 227 KB) [file 10067_2022_6402_MOESM1_ESM.docx]

**Supplemental Figure 1:** PRISMA 2020 flow diagram

**Identification of studies via databases and registers**

Records removed *before screening*:

Duplicate records removed (n=124)

Records marked as ineligible by automation tools (n =0)

Records removed for other reasons (n =0)

Records identified (n=515) from

PubMed (n = 74)

Web of Science (n = 65)

Scopus (n = 346)

Cochrane (n = 30)

Register (n = 0)

**Identification**

Records screened

(n = 391)

Records excluded**

(n = 375)

Reports sought for retrieval

(n =16)

Reports not retrieved

(n =0)

**Screening**

Reports excluded:

Incorrect study design (n =5)

Duplicate (n =1)

Reports assessed for eligibility

(n =16)

Studies included in review

(n = 16)

Reports of included studies

(n = 16)

**Included**

*From:*  Page MJ, McKenzie JE, Bossuyt PM, Boutron I, Hoffmann TC, Mulrow CD, et al. The PRISMA 2020 statement: an updated guideline for reporting systematic reviews. BMJ 2021;372:n71. doi: 10.1136/bmj.n71

**Supplemental Table 2**: Osteoarthritis related markers outcomes

| **Study** | **Biomarkers** | **Conclusion** |  |
| --- | --- | --- | --- |
| ***OA related biochemical markers*** | | | |
| Srivastava R (2018) | Serum COMP | Serum COMP level in COL+ paracetamol arm remains stable over two years of follow-up as compared to paracetamol arm. |  |
| Leung YY (2018) | Serum hs-CRP; sf IL-6, 8, 18; sf TNFα; sf CD14, sf CTX-I, II; urinary CTXII/Cr | Mean levels of serum hs-CRP and SF CTX-I were significantly reduced in the colchicine but not the placebo-treated arm. Mean levels of sf IL-6, IL-8, TNFα, CD14, and IL-18 were all reduced in the colchicine arm, but not significantly. |  |
| Davis CR (2020) | CRP, CK, ALT, AST | No significant differences for CRP, CK, or liver enzymes between groups. |  |
| ***OA related imaging markers*** | | | |
| Leung YY (2018) | MRI-assessed Effusion and infrapatellar synovitis | Among a sub-set of patients (n=20) how had MRI at baseline, no statistically significant changes in effusion size or infrapatellar synovitis were noted in either treatment arm over the study period. |  |
| Davis CR (2020) | Ultrasound-assessed synovitis | No significant differences in ultrasound assessed synovitis grade. |  |
| ALT: alanine aminotransferase; AST: aspartate aminotransferase; CK: creatinine kinase; COMP: cartilage oligomeric matrix protein; CRP: c-reactive protein; CTXI, II: C-terminal cross-linked telopeptides of type I collagen, Type II; IL: interleukin; sf: synovial fluid; hs-CRP: high sensitive C-reactive protein; TNFα: tumour necrosis factorα; NR; not reported | | | |

**Supplemental Table 2**: Knee osteoarthritis related quality of life outcomes

| **Study** | **PRO instrument** | **Conclusion** |
| --- | --- | --- |
| Das SK (2002) AC | ModHAQ | The ModHAQ scores were improved significantly in the COL group compared to control at 5-months follow-up. |
| Das SK (2002) OC | ModHAQ | The ModHAQ scores were improved significantly in the COL group compared to control at 4- and 5-months follow-up. |
| Leung YY (2018) | HAQ; SF-36 PCS and MCS | No statistically significant changes in HAQ or SF-36 (PCS and MCS) was noted in either treatment arm over the study period. |
| AC: Arthritis Care & Research; COL: colchicine; HAQ: Health Assessment Questionnaire; OC: Osteoarthritis and Cartilage; PRO: patients reported outcome; SF-36 PCS: physical summary score of SF-36; SF-36 MCS: mental summary score of SF-36; ModHAQ; Modified Clinical Health Assessment Questionnaire; ModHAQ: a non-validated modification of a scale used at All India Institute of Medical Sciences, New Delhi and ClinHAQ scale | | |

**Supplemental Table 3**: Sensitivity analysis for pooled standardised mean difference for change in osteoarthritis associated pain

| **Study removed** | **Effect size, SMD (95%CI)** |
| --- | --- |
| *Leung 2018* | -0.25 (0.72, 0.22) |
| *Erden 2021* | -0.22 (-0.69, 0.26) |
| *Davis 2020* | -0.29 (-0.67, 0.09) |
| *Das 2002 OC* | -0.11 (-0.55, 0.32) |
| *Das 2002 AC* | -0.05 (-0.42, 0.32) |
| *Amirpour2016* | -0.08 (-0.49, 0.33) |
| **Overall effect estimate** | -0.17 (-0.55, 0.22) |

**Supplemental Table 4**: Sensitivity analysis for pooled standardised mean difference for change in osteoarthritis associated disfunction

| **Study removed** | **Effect size, SMD (95%CI)** |
| --- | --- |
| Das 2002 AC | -0.24 (-0.91, 0.42) |
| *Das 2002 OC* | -0.27 (-1.00, 0.46) |
| *Erden 2021* | -0.65 (-1.11, -0.18) |
| **Overall effect estimate** | -0.37 (-0.87, 0.13) |

**Supplemental Figure 2:** Sub-group analysis including studies with placebo as a comparator


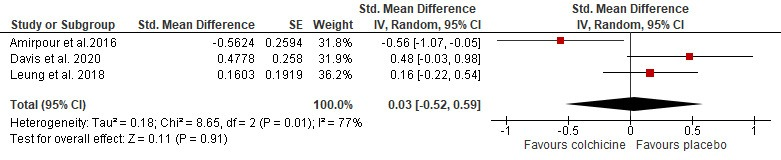


**Supplemental Table 5:** List of ongoing colchicine trials in OA patients

| **S.No** | **Registration Numbers** | **Title** | **Acronym** | **Status** | **Study Results** | **Conditions** | **Interventions** | **Outcome Measures** | **Age** | **Phases** | **Enrollment** | **Study Designs** | **Start Date** | **Country** |
| --- | --- | --- | --- | --- | --- | --- | --- | --- | --- | --- | --- | --- | --- | --- |
| 1 | NCT04601883; EUCTR2020-002803-20-DK; | COL as Treatment for People With Hand OA | COLOR | Recruiting | No Results Available | OA Hand\|OA Finger | Drug: COL 0.5 MG\|Drug: PLB | Finger pain\|Function\|Thumb base pain\|Pain hands\|Joint activity\|Patient global assessment\|Quality of life measured by European Quality of Life 5 Dimensions\|Hand strength\|Number of treatment responders according to OMERACT-OARSI response criteria | 18 Years and older   (Adult, Older Adult) | Phase 4 | 100 | Allocation: Randomized\|Intervention Model: Parallel Assignment\|Masking: Quadruple (Participant, Care Provider, Investigator, Outcomes Assessor)\|Primary Purpose: Treatment | 15/01/2021 | Denmark |
| 2 | NCT03913442 | COL for the Treatment of OA of the Knee | CLOAK | Recruiting | No Results Available | OA\|Osteo Arthritis Knee | Drug: COL 0.8 mg or 0.6 mg orally once daily\|Drug: PLB oral capsule | Difference in mean changes of VAS pain scores between treatment groups\|Mean changes and absolute differences in the Knee Injury and OA Outcome Score (KOOS) for pain\|mean changes of KOOS stiffness\|mean absolute doses and changes in dosage of acetaminophen or other medications used for pain between and within the groups at baseline and 3 months.\|mean changes of KOOS physical function\|mean changes between total KOOS scores between and within the groups at 3 months. | 18 Years to 99 Years   (Adult, Older Adult) | Phase 4 | 120 | Allocation: Randomized\|Intervention Model: Parallel Assignment\|Masking: Double (Participant, Care Provider)\|Primary Purpose: Treatment | 15/05/2019 | US |
| OA: osteoarthritis; COL: colchicine; PLB: placebo  Searched from: Clinicaltrial.gov from <https://clinicaltrials.gov/> on 17 Feb 2022; International Clinical Trial Registry Platform from [https://trialsearch.who.int/Default.aspx on 22 Feb 2022](https://trialsearch.who.int/Default.aspx%20on%2022%20Feb%202022); EU Clinical Trial Register from <https://www.clinicaltrialsregister.eu/ctr-search/search> on 22 Feb 2022 | | | | | | | | | | | | | | |

**Search strategies**

**PRISMA 2020 checklist**

| **Section and Topic** | **Item #** | **Checklist item** | **Location where item is reported** |
| --- | --- | --- | --- |
| **TITLE** | | |  |
| Title | 1 | Identify the report as a systematic review. | 1 |
| **ABSTRACT** | | |  |
| Abstract | 2 | See the PRISMA 2020 for Abstracts checklist. | 1 |
| **INTRODUCTION** | | |  |
| Rationale | 3 | Describe the rationale for the review in the context of existing knowledge. | 1-4 |
| Objectives | 4 | Provide an explicit statement of the objective(s) or question(s) the review addresses. | 1-4 |
| **METHODS** | | |  |
| Eligibility criteria | 5 | Specify the inclusion and exclusion criteria for the review and how studies were grouped for the syntheses. | 4-6 |
| Information sources | 6 | Specify all databases, registers, websites, organisations, reference lists and other sources searched or consulted to identify studies. Specify the date when each source was last searched or consulted. | 4-6  Supplement  Protocol |
| Search strategy | 7 | Present the full search strategies for all databases, registers and websites, including any filters and limits used. | 4-6  Supplement  Protocol |
| Selection process | 8 | Specify the methods used to decide whether a study met the inclusion criteria of the review, including how many reviewers screened each record and each report retrieved, whether they worked independently, and if applicable, details of automation tools used in the process. | 4-6  Protocol |
| Data collection process | 9 | Specify the methods used to collect data from reports, including how many reviewers collected data from each report, whether they worked independently, any processes for obtaining or confirming data from study investigators, and if applicable, details of automation tools used in the process. | 4-6  Protocol |
| Data items | 10a | List and define all outcomes for which data were sought. Specify whether all results that were compatible with each outcome domain in each study were sought (e.g. for all measures, time points, analyses), and if not, the methods used to decide which results to collect. | 4-6  Protocol |
|  | 10b | List and define all other variables for which data were sought (e.g. participant and intervention characteristics, funding sources). Describe any assumptions made about any missing or unclear information. | 4-6  Protocol |
| Study risk of bias assessment | 11 | Specify the methods used to assess risk of bias in the included studies, including details of the tool(s) used, how many reviewers assessed each study and whether they worked independently, and if applicable, details of automation tools used in the process. | 4-6  Protocol |
| Effect measures | 12 | Specify for each outcome the effect measure(s) (e.g. risk ratio, mean difference) used in the synthesis or presentation of results. | 4-6  Protocol |
| Synthesis methods | 13a | Describe the processes used to decide which studies were eligible for each synthesis (e.g. tabulating the study intervention characteristics and comparing against the planned groups for each synthesis (item #5)). | 5-6  Protocol |
|  | 13b | Describe any methods required to prepare the data for presentation or synthesis, such as handling of missing summary statistics, or data conversions. | 5-6  Protocol |
|  | 13c | Describe any methods used to tabulate or visually display results of individual studies and syntheses. | 5-6  Protocol |
|  | 13d | Describe any methods used to synthesize results and provide a rationale for the choice(s). If meta-analysis was performed, describe the model(s), method(s) to identify the presence and extent of statistical heterogeneity, and software package(s) used. | 5-6  Protocol |
|  | 13e | Describe any methods used to explore possible causes of heterogeneity among study results (e.g. subgroup analysis, meta-regression). | 4-6  Protocol |
|  | 13f | Describe any sensitivity analyses conducted to assess robustness of the synthesized results. | 6  Protocol |
| Reporting bias assessment | 14 | Describe any methods used to assess risk of bias due to missing results in a synthesis (arising from reporting biases). | 5-6  Protocol |
| Certainty assessment | 15 | Describe any methods used to assess certainty (or confidence) in the body of evidence for an outcome. | NA |
| **RESULTS** | | |  |
| Study selection | 16a | Describe the results of the search and selection process, from the number of records identified in the search to the number of studies included in the review, ideally using a flow diagram. | 6-9  Supplement |
|  | 16b | Cite studies that might appear to meet the inclusion criteria, but which were excluded, and explain why they were excluded. | 6-9  Supplement |
| Study characteristics | 17 | Cite each included study and present its characteristics. | 6-9  Supplement |
| Risk of bias in studies | 18 | Present assessments of risk of bias for each included study. | 6-9  Supplement |
| Results of individual studies | 19 | For all outcomes, present, for each study: (a) summary statistics for each group (where appropriate) and (b) an effect estimate and its precision (e.g. confidence/credible interval), ideally using structured tables or plots. | 6-9  Supplement |
| Results of syntheses | 20a | For each synthesis, briefly summarise the characteristics and risk of bias among contributing studies. | 6-9  Supplement |
|  | 20b | Present results of all statistical syntheses conducted. If meta-analysis was done, present for each the summary estimate and its precision (e.g. confidence/credible interval) and measures of statistical heterogeneity. If comparing groups, describe the direction of the effect. | 6-9  Supplement |
|  | 20c | Present results of all investigations of possible causes of heterogeneity among study results. | 6-9  Supplement |
|  | 20d | Present results of all sensitivity analyses conducted to assess the robustness of the synthesized results. | 7  Supplement |
| Reporting biases | 21 | Present assessments of risk of bias due to missing results (arising from reporting biases) for each synthesis assessed. | 6-9  Supplement |
| Certainty of evidence | 22 | Present assessments of certainty (or confidence) in the body of evidence for each outcome assessed. | 6-9  Supplement |
| **DISCUSSION** | | |  |
| Discussion | 23a | Provide a general interpretation of the results in the context of other evidence. | 8-10 |
|  | 23b | Discuss any limitations of the evidence included in the review. | 8-10 |
|  | 23c | Discuss any limitations of the review processes used. | 8-10 |
|  | 23d | Discuss implications of the results for practice, policy, and future research. | 8-10 |
| **OTHER INFORMATION** | | |  |
| Registration and protocol | 24a | Provide registration information for the review, including register name and registration number, or state that the review was not registered. | 1 |
|  | 24b | Indicate where the review protocol can be accessed, or state that a protocol was not prepared. | 1  Supplement |
|  | 24c | Describe and explain any amendments to information provided at registration or in the protocol. | NA |
| Support | 25 | Describe sources of financial or non-financial support for the review, and the role of the funders or sponsors in the review. | 11 |
| Competing interests | 26 | Declare any competing interests of review authors. | 11 |
| Availability of data, code and other materials | 27 | Report which of the following are publicly available and where they can be found: template data collection forms; data extracted from included studies; data used for all analyses; analytic code; any other materials used in the review. | NA |
